# Humidity Response of Cellulose Thin Films

**DOI:** 10.1021/acs.biomac.1c01446

**Published:** 2022-02-28

**Authors:** David Reishofer, Roland Resel, Jürgen Sattelkow, Wolfgang J. Fischer, Katrin Niegelhell, Tamilselvan Mohan, Karin Stana Kleinschek, Heinz Amenitsch, Harald Plank, Tekla Tammelin, Eero Kontturi, Stefan Spirk

**Affiliations:** † Institute of Bioproducts and Paper Technology, Graz University of Technology, Inffeldgasse 23, Graz 8010, Austria; ‡ Institute for Solid State Physics, Graz University of Technology, Petersgasse 16, Graz 8010, Austria; § Institute for Electron Microscopy and Nanoanalysis, Graz University of Technology, Steyrergasse 17, Graz 8010, Austria; ∥ Institute of Chemistry and Technology of Biobased Systems, Graz University of Technology, Stremayrgasse 9, Graz 8010, Austria; ⊥ Institute for Inorganic Chemistry, Graz University of Technology, Stremayrgasse 9, Graz 8010, Austria; # High Performance Fibre Products, VTT Technical Research Center of Finland Ltd, Espoo FI-02044 VTT, Finland; ∇ Department of Bioproducts and Biosystems, School of Chemical Technology, Aalto University, Espoo 02150, Finland

## Abstract

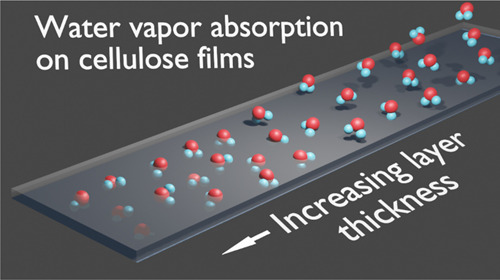

Cellulose–water interactions are crucial to understand biological processes as well as to develop tailor made cellulose-based products. However, the main challenge to study these interactions is the diversity of natural cellulose fibers and alterations in their supramolecular structure. Here, we study the humidity response of different, well-defined, ultrathin cellulose films as a function of industrially relevant treatments using different techniques. As treatments, drying at elevated temperature, swelling, and swelling followed by drying at elevated temperatures were chosen. The cellulose films were prepared by spin coating a soluble cellulose derivative, trimethylsilyl cellulose, onto solid substrates followed by conversion to cellulose by HCl vapor. For the highest investigated humidity levels (97%), the layer thickness increased by ca. 40% corresponding to the incorporation of 3.6 molecules of water per anhydroglucose unit (AGU), independent of the cellulose source used. The aforementioned treatments affected this ratio significantly with drying being the most notable procedure (2.0 and 2.6 molecules per AGU). The alterations were investigated in real time with X-ray reflectivity and quartz crystal microbalance with dissipation, equipped with a humidity module to obtain information about changes in the thickness, roughness, and electron density of the films and qualitatively confirmed using grazing incidence small angle X-ray scattering measurements using synchrotron irradiation.

## Introduction

The interaction of water vapor with surfaces represents one of the crucial aspects to be considered in technology development, exploitation, and product engineering.^1^ This is particularly prominent with soft materials like polymers because vapor can penetrate the chain network, altering its properties. Indeed, the control over water vapor migration through or into a material (e.g., a membrane or a film) is pivotal in many cases when realizing or triggering certain materials characteristics. For synthetic hydrophobic polymers, the vapor transport is often straightforward to monitor, model, and control.^2^ The major interactions comprise diffusion into and out of the polymer as well as adsorption/desorption phenomena.^3^ For biopolymers, however, the case is more complicated. They usually form hydrophilic or amphiphilic, porous networks that swell considerably when exposed to water vapor, rendering the solution-diffusion model inapplicable.^4^ One of the more intricate cases with biopolymers is cellulose, the main ingredient of all plants. Cellulose forms highly specific semicrystalline microfibrils which are further organized into complex hierarchical superstructures in plant fibers. Water interactions are highly relevant for the fibers in their native growth environment^5^ and they are equally important for the manifold applications of cellulose fibers^6−8^ as well as for modern usages designed for various nanocellulose grades.^9−13^ In this realm, many studies exist on the vapor transport mechanisms in macroscopic products prepared from cellulose fibers, such as paper and textiles or regenerated films and fibers.^3,14−19^ In addition, commendable efforts have been undertaken to model the vapor transport through certain cellulose-based structures.^20−22^ Such studies are generally driven by industrial applications and they are specific to the relevant macroscopic structures where a multiscale morphology plays a significant role.^23^ Besides the pulp and paper industry, emerging fields include nanocellulose-based optoelectronic devices,^24−26^ sensors,^27,28^ and medicine.^29^

In this fundamental contribution, we aim at minimizing the morphological contribution by monitoring the water vapor interactions in homogeneous, two-dimensionally confined ultrathin films of highly amorphous cellulose. This way, we can gain fundamental information on the influence of various industrially relevant treatments on the vapor uptake of cellulose and these results are not obfuscated with the morphology factor. The treatments prior to water vapor uptake measurements comprise drying (105 °C for 1 h), swelling, and swelling/drying (105 °C for 1 h). The films were prepared from trimethylsilyl cellulose (TMSC) which was regenerated into cellulose after film deposition by spin coating. Two different TMSC grades were employed, featuring different solubility because of a difference in the degree of substitution. Moreover, the film structure was tuned by the use of two different solvents (chloroform and THF) in the spin coating step. The surface morphology of the films was characterized by atomic force microscopy (AFM) and the mechanical properties (stiffness) by nanoindentation. The water vapor uptake was followed by in situ X-ray reflectivity (XRR), quartz crystal microbalance with dissipation monitoring (QCM-D), and grazing incidence small angle scattering (GI-SAXS). This study is related to recently published studies on the water uptake of various cellulose thin films^22,30−33^ but here the approach is more revealing for the molecular arrangements of water molecules inside homogeneous cellulose layers with subtle systematic variations. The results revealed a complex ordering of water to, at times, three different layers within the film, laying the groundwork for the profound understanding of vapor-cellulose interactions and their explicit utilization in modern applications.

## Materials and Methods

### Materials

TMSC (from MCC, DS: 2.7–2.9, *M*_w_: 130 kDa; from spruce pulp, DS: 2.0, *M*_w_: 120 kDa), obtained from TITK (Rudolstadt, Germany) was used as the starting material for the thin film preparation. Hydrochloric acid (37 wt %), chloroform (99 wt %), THF (99 wt %), and sulfuric acid (95 wt %) were purchased from VWR chemicals and hydrogen peroxide (30 wt %) from Sigma-Aldrich. All chemicals were used without purification. Silicon wafer and gold QCM-D sensors were purchased from Q-Sense, AB, Gothenburg, Sweden (fundamental resonance frequency, *f*_0_ = 5 MHz; sensitivity constant, *C* = −0.177 mg·m^–2^·Hz^–1^), and Filter Chromafil Xtra PVDF-45/25 0.45 μm and petri dishes (20 mL; 5 cm diameter) were used as obtained.

### Film Preparation

The silicon wafer substrates (native oxide layer, 1.4 × 1.4 cm^2^) for the XRR and GI-SAXS measurements were cleaned with “piranha” acid (H_2_SO_4_:H_2_O_2_ = 7:3 (v/v)) for 30 min and neutralized afterward with distilled water. QCM-D gold quartz crystals were cleaned with a UV ozone cleaner (Bioforce Nanosciences Inc., California, USA) for a minimum of 20 min. For the preparation of the cellulose thin films, two different TMSCs (TMSC_A_, from MCC, 2.7–2.9; TMSC_S_, from spruce, DS: 2.0) were employed and dissolved in chloroform (TMSC_A_: 15 mg·mL^–1^) and tetrahydrofuran (TMSC_S_: 9 mg·mL^–1^). Afterward, the solutions were filtered and used to prepare thin films via spin coating (4000 rpm, 2500 rpm·s^–1^, 60 s) on QCM-D gold quartz crystals and silicon wafers. The film thickness of the TMSC films was approximately 150 nm. In the next step, the films were regenerated using 12 wt % HCl vapor for 12 min.^34^ After the regeneration, the cellulose samples were subjected to different treatments: (i) drying at 105 °C for 1 h, (ii) swelling with deionized H_2_O for 30 min, and (iii) swelling with deionized H_2_O for 30 min followed by drying at 105 °C for 1 h.

### AFM

Measurements were performed with a FastScanBio platform operated by a Nanoscope V controller (Bruker Nano Surface Offices, Santa Barbara, CA). Nanomechanical characterization was executed in PeakForce-mode providing additional information on Young’s modulus, sample adhesion, energy dissipation, and surface deformation with laterally resolved character. All measurements were performed in an air-conditioned environment (21 °C) under an acoustical enclosure box. A RTESPA-300 (Bruker AFM Probes, Camarillo, CA) cantilever with nominal spring constants of 40 N/m was used in all experiments. Calibration was done for each tip using the calibration kit PFQNM-SMPKIT-12 M (Bruker AFM Probes, Camarillo, CA). Deflection sensitivity was ramped against sapphire, and the cantilever spring constant was evaluated by thermal tune, and tip end radii were estimated via a defined titanium-oxide roughness sample. First TMSC and cellulose samples from spin coating and post-treatment were carefully scratched with a sharp razor blade to create a mark in the layer with silicon oxide as level zero. At least four different areas per sample have been investigated with minimum two measurements at the scratched edges and at top layer positions, each. Experimental parameters were optimized to obtain stable imaging conditions with the lowest possible energy dissipation and sample deformation.

### XRR

XRR measurements were performed using a PANalytical Empyrean goniometer system with radiation produced by a copper sealed tube (λ = 0.154178 nm). The primary side of the reflectometer was equipped with a 20 mm beam mask, a multilayer mirror, a 1/32° slit, and an automatic beam attenuator. On the secondary side, a receiving slit of 0.1 mm and a Soller slit of 0.02 rad were used in front of the PANalytical PIXEL3D point detector. The sample stage was a domed DHS 900 from Anton Paar,^35^ equipped with a SHT15 humidity sensor to monitor the relative humidity (RH) and the temperature during the in situ swelling measurements. The RH was controlled using a S-503 humidity generator from Michell instruments. For each humidity step, an equilibration time of 30 min was accomplished. XRR measurements were performed in the 2θ region 0.030–9.999° with a step size of 0.006°. The evaluation of the data was performed with the X’Pert Reflectivity (Panalytical, C_6_H_10_O_5_ for cellulose was used) software package providing information on the electron density, layer thickness, and the roughness of the films by applying Parrat^36^ formalism and the disturbance term of Nevot–Croce.^37^ The fitting procedures yielded stable fits with errors being below 0.3%.

### QCM-D

Water vapor absorption experiments were carried out in a QCM-D (Q-Sense, AB, Gothenburg, Sweden) equipped with a humidity module (QHM 401). The frequencies of the pure QCM-D sensor crystal and the spin-coated starting areal mass were determined in air. At the beginning of the water vapor absorption experiments, the samples were allowed to equilibrate at 11 %RH (saturated LiCl solution) for 18 h to obtain a stable baseline. For the following humidity steps, stable values were adjusted by a suitable salt solution (11 → 33 → 53 → 75 → 97 %RH) after 30 min of equilibration (100 μL/min at 23 °C). For the highest humidity level (97 %RH) equilibration was done for 45 min. More information on the used salts can be found in the Supporting Information. The collected frequency data were stitched together using QTools Software and the areal mass as well as the film thickness were calculated according to the Sauerbrey equation:
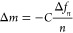
1where *n* is the measurement overtone number (*n* = 1, 3, 5, 7, ...), Δ*f_n_* = *f_n_* – *f*_0_ is the resonance frequency, and *C* is the sensitivity constant of the sensor. For the calculation of the film thickness of the samples, the individual starting area mass (Δ*f*_3_) of the samples and the calculated densities of the XRR measurements were used. The samples were stored in a desiccator to protect them from environmental influences and taken out 15 min before the measurement started.

### GI-SAXS

The in situ GI-SAXS experiments were performed at the high-flux SAXS beamline at Elettra synchrotrone in Trieste, Italy, with an X-ray energy of 8 keV (λ = 1.54 nm). The sample stage was a domed DHS 900 from Anton Paar. As the detection system, a 2D Pilatus3 1 M Detector System from Dectris was used. The sensitive area is 981 × 1043 pixels with a pixel size of 172 × 172 μm^2^. As the calibration standard, silver behenate with a lamellar spacing of 58.38 Å was used. The sample-to-detector distance was determined to be 1911.5 mm and the incidence angle was set to 0.35°. For the generation of the RH, an S-503 humidity generator from Michell instruments was used. For the data analysis, horizontal cuts at the position of the Yoneda peak have been calculated. In order to determine the diffuse scattering of the hierarchical structure beyond the resolution limit as well as the background level and the surface roughness, a fit function was used; eqs 2 and 3:
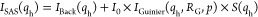
2

with
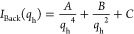
3where *q*_h_ denotes the in-plane scattering vector, *I*_Back_ (with *A*, *B*, *C* fitting coefficients) accounting for background, the diffuse scattering of the hierarchical structure beyond the resolutions limit, and surface roughness, respectively.

*I*_Guinier_ is a simplified Guinier–Porod Model to determine the Guinier and Porod coefficients^38^ and *S*(*q*_h_) refers to a simplified structure factor using a Lorentzian peak with *I*_p_ intensity, *q*_p_ position, and σ_p_ width for the first order correlation peak (eq 4).
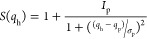
4

For the calculation of the *d*-spacing, the contribution of the vertical scattering vector has been taken into account (*d*_spacing_ = 2π/*q* and the estimation of *d*_max_ = 2π/*q*).

## Results and Discussion

A very common procedure to prepare cellulose ultrathin films is to employ acid labile TMSC which is deposited by spin coating onto silicon wafers and subsequently exposed to HCl vapors. This exposure cleaves off the silyl groups leaving a rather amorphous cellulose thin film with only short-range ordered cellulose molecules.^39^ Since the vapor pressure of the solvent during spin coating can affect the film structure, two different TMSC derivatives featuring a different solubility behavior (CHCl_3_ and THF) and molecular mass (*M*_w_: 130 vs 120 kDa) were selected to prepare cellulose thin films. In the following, the cellulose films derived from TMSC_A_ are denoted as Cell_A_ whereas those from TMSC_S_ are denoted as Cell_S_.

**Figure 1:**
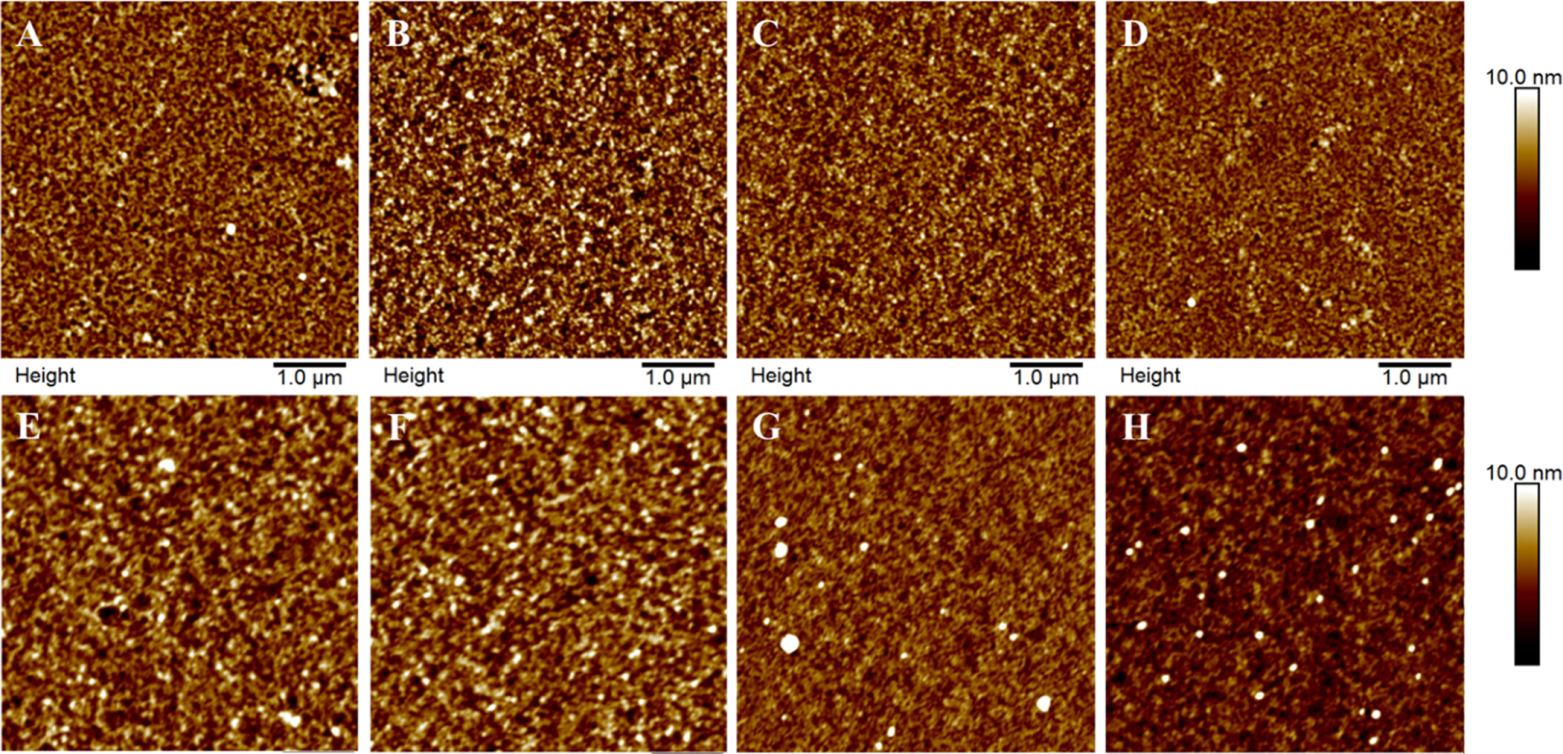
AFM topography images (5 × 5 μm^2^) of the differently prepared cellulose films before and after the different treatments. (A–D) Cell_A_, (A) nontreated, (B) dried, (C) swollen, (D) swollen/dried, (E–G) Cell_S_ (E) nontreated, (F) dried, (G) swollen, (H) swollen/dried).

The AFM images of the two differently prepared cellulose films including the various treatments are depicted in Figure 1. The morphology of the surfaces was featureless. The roughness for all the films was similar (1.2–1.6 nm), with outliers being the dried Cell_A_ (2.1 ± 0.1 nm) and the swollen Cell_S_ (1.1 ± 0.1) sample (Table 1).

**Table 1:** Comparison of Surface Roughness Determined by AFM and XRR^a^

	Cell_A_			Cell_S_		
	XRR	AFM		XRR	AFM	
	RMS [nm]	RMS [nm]	stiffness [GPa]	RMS [nm]	RMS [nm]	stiffness [GPa]
nontreated	1.6 ± 0.1	1.5 ± 0.1	4.0 ± 1.2	1.5 ± 0.1	1.5 ± 0.1	5.5 ± 0.5
dried	2.8 ± 0.2	2.1 ± 0.1	4.9 ± 0.8	1.7 ± 0.1	1.6 ± 0.1	6.0 ± 0.5
swollen	1.6 ± 0.1	1.5 ± 0.1	4.5 ± 1.3	1.7 ± 0.1	1.1 ± 0.1	6.0 ± 0.5
swollen/dried	1.7 ± 0.1	1.4 ± 0.1	4.8 ± 1.0	1.4 ± 0.1	1.2 ± 0.1	6.0 ± 0.5

a Average stiffness determined by AFM is shown.

Furthermore, AFM nanoindentation experiments were performed. Calibration of these measurements was performed using a calibration kit and reference measurements at a scratch in the thin films giving the silicon substrate as a reference surface, allowing for obtaining quantitatively comparable data (Table 1). Cell_S_ samples exhibited higher stiffnesses than the Cell_A_, whereas the nontreated films exhibited the largest discrepancy with 4.0 ± 1.2 and 5.5 ± 0.5 GPa, respectively. For both film types, the nontreated samples displayed a lower stiffness than those which had been subjected to different treatments. For Cell_A_, the treated films were in a range from 4.5 ± 1.3 (swollen) to 4.9 ± 0.8 GPa (dried), while for Cell_S_ the stiffness did not significantly vary for the differently treated samples (5.9–6.0 ± 0.5 GPa). However, the alteration in stiffness for the different films should not be overinterpreted as the error bar intervals overlap for all measurements; even for the nontreated Cell_A_ and Cell_S_ films where the difference is the largest among all samples.

**Figure 2:**
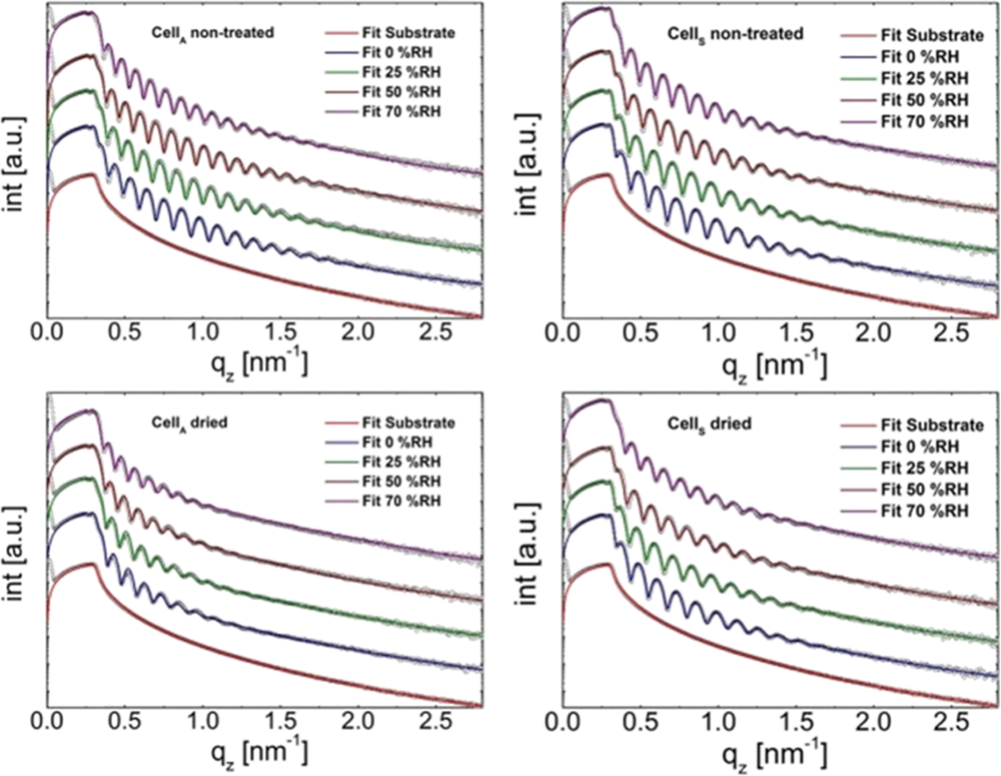
XRR curves and corresponding layer fit of the two different cellulose films (Cell_A_, left column; Cell_S_, right column) samples.

The water vapor uptake was first monitored by XRR since it provides insights into changes in film thickness, density, as well as on the roughness of the films. The obtained XRR curves and the corresponding layer fits are shown in Figure 2 for the nontreated and the dried samples. More data are available in the Supporting Information (Figures S1 and S2).

The XRR data revealed that a multilayer approach with varying densities of the respective layers was required to fit the data. Such multilayer fittings can be physically related to the different mass densities of films at the respective interfaces (e.g., cellulose–substrate or cellulose–air).^40^ As a consequence, a local statistical density distribution was obtained as already shown earlier.^41^ For all the Cell_A_ samples except the preswollen sample, a two-layer model fit and for the Cell_S_ ones, a three-layer model fit yielded excellent agreement between the data and the fit. Similar to the AFM data, also the XRR results revealed some differences between the two different cellulose samples.

**Figure 3:**
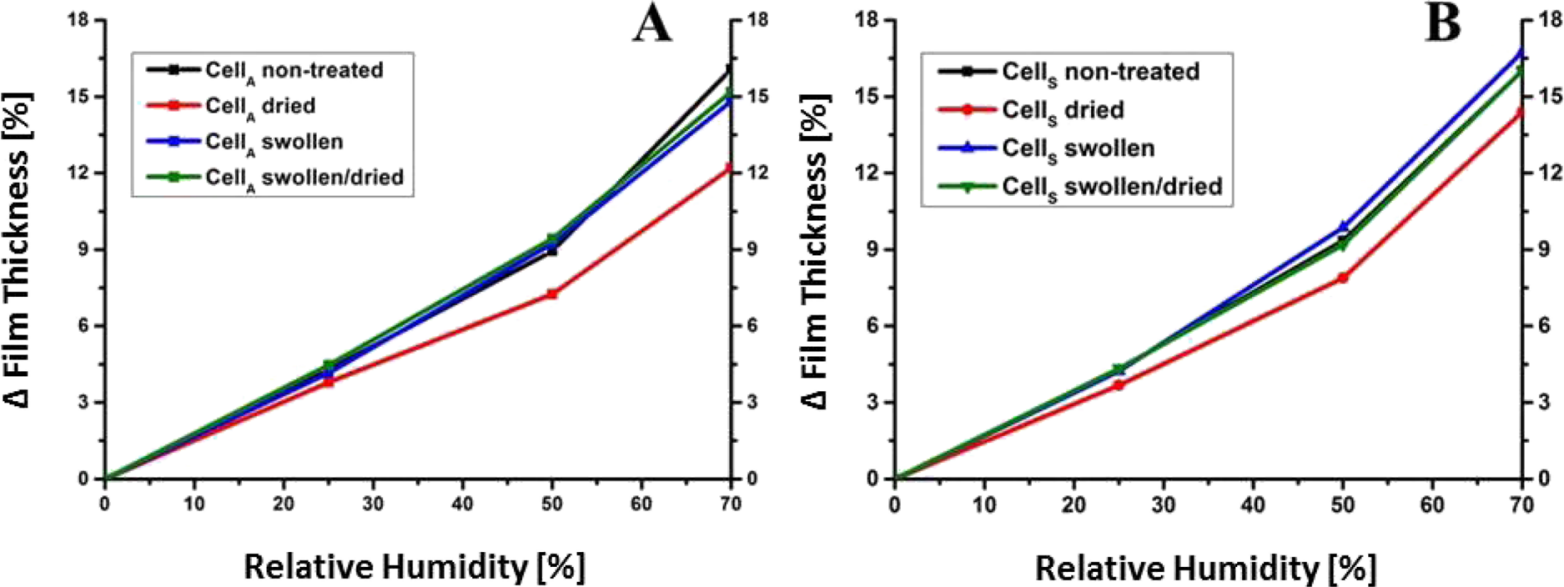
Film thickness increase of cellulose thin films with different treatments at various humidity levels determined by XRR measurements during the water vapor uptake process. (A) Cell_A_ samples (B) Cell_S_ samples.

The XRR investigations (for comprehensive data see Tables S1 and S2; Supporting Information) revealed that the initial film thickness at 0%RH of the Cell_A_ samples is slightly higher (51 ± 3 nm) than those of Cell_S_ (43 ± 2 nm). As the RH increases, the fringes of the cellulose film shifted to a lower *q*_z_ indicating that the films start to incorporate water vapor thereby increasing the film thickness. This is in line with previous reports using ellipsometry.^30^ At 25%RH, all Cell_A_ samples exhibited a similar relative increase in layer thickness (3.8–4.5%) independent of whether they had been subjected to treatments or not (Figure 3). However, at 50%RH alterations, the behavior of the differently treated samples started to evolve. The dried samples for instance were prone to a lower water vapor uptake compared to the other films (7.2 vs 9.0–9.5% thickness increase). This behavior was even more pronounced at 70%RH where the dried films featured a relative film thickness increase of 12.2% whereas the other samples exhibited a higher relative increase (14.6–16.1%). The Cell_S_ samples (Figure 3B) displayed the same trends. While the water vapor uptake of the nontreated films equaled the Cell_A_ films, the extent of the water vapor uptake for the swollen films was rather high, particularly at high RH. For instance, the swollen Cell_S_ samples exhibited a relative thickness increase of 16.8% while for the Cell_A_ samples only 14.6% increase was observed. Another remarkable difference was the lower impact of heating on the water vapor uptake capacity even at high RH for the Cell_S_ samples (comp. at 70%RH, 14.4 vs 12.2% relative increase).

An appealing feature of XRR measurements in these experiments is to obtain roughness values as a function of RH. Surprisingly, the impact of humidity on the surface roughness of the films was rather low and just slight changes in the range of max. 0.3 nm were noticeable. As for the AFM studies, the dried Cell_A_ samples exhibited the highest roughness (2.8 nm at 0%RH). For the Cell_S_ samples, similar trends were observed albeit the films were slightly smoother and also the difference in roughness to the heat-treated films was not as pronounced (1.5 vs 1.7 nm for nontreated vs dried; Table S2, and Figure S1, Supporting Information). As mentioned above, the cellulose layer was fitted by a two and three-layer model, depending on the type of cellulose. This fitting was necessary as in the case of only a single cellulose layer, a suitable fit with the data could not be obtained. For all the samples, a layer at the substrate interface had to be introduced to result in a very good fit. The thickness of this cellulose layer between the substrate and the cellulose “bulk” was in a range between 0.5 and 0.7 nm at 0%RH for all films (Table S2, Supporting Information), corresponding to one or two stapled cellulose molecules (compare thickness of graphene monolayers: 0.3 nm). The impact of humidity on this layer in terms of thickness was proven to be negligible for most cases and hardly any variations could be observed. Further, the density of this layer for the nontreated films indicated that the rather rigid cellulose molecules were not able to perfectly cover the whole SiO_2_ surface.

For the Cell_S_ samples and the preswollen Cell_A_ sample, the inclusion of an additional layer was required which reflected alterations at the cellulose–air interface. For those samples, even a two-layer fit did not yield satisfying results. This third layer had a thickness in the range 3.7–5.2 nm and showed a decreased density (1.0–1.2 g·cm^–3^ at 0%RH) compared to the bulk cellulose layer. These results are in good agreement with a recent study on similar thin films analyzed by surface plasmon resonance spectroscopy that revealed the presence of a surface layer that has different properties than the bulk film.^40^

The incorporation of water vapor into the film structure can influence the mass density in two ways. First, the filling of gaps, i.e., replacement of air against water will increase the density of the films. Second, if the water was directly incorporated into the cellulose supramolecular structure, the resulting electron density should be smaller than that of the cellulose itself. The mass density for most of the cellulose “bulk” layers as determined by XRR is in the range for amorphous cellulose (1.48 g·cm^–3^).^42,43^ Accordingly, the vapor uptake leads to decreasing density of most of the films by increasing humidity levels (Table 1). Densities decrease down to 1.35 g·cm^–3^ for both samples at humidity levels of 70%RH.

**Figure 4:**
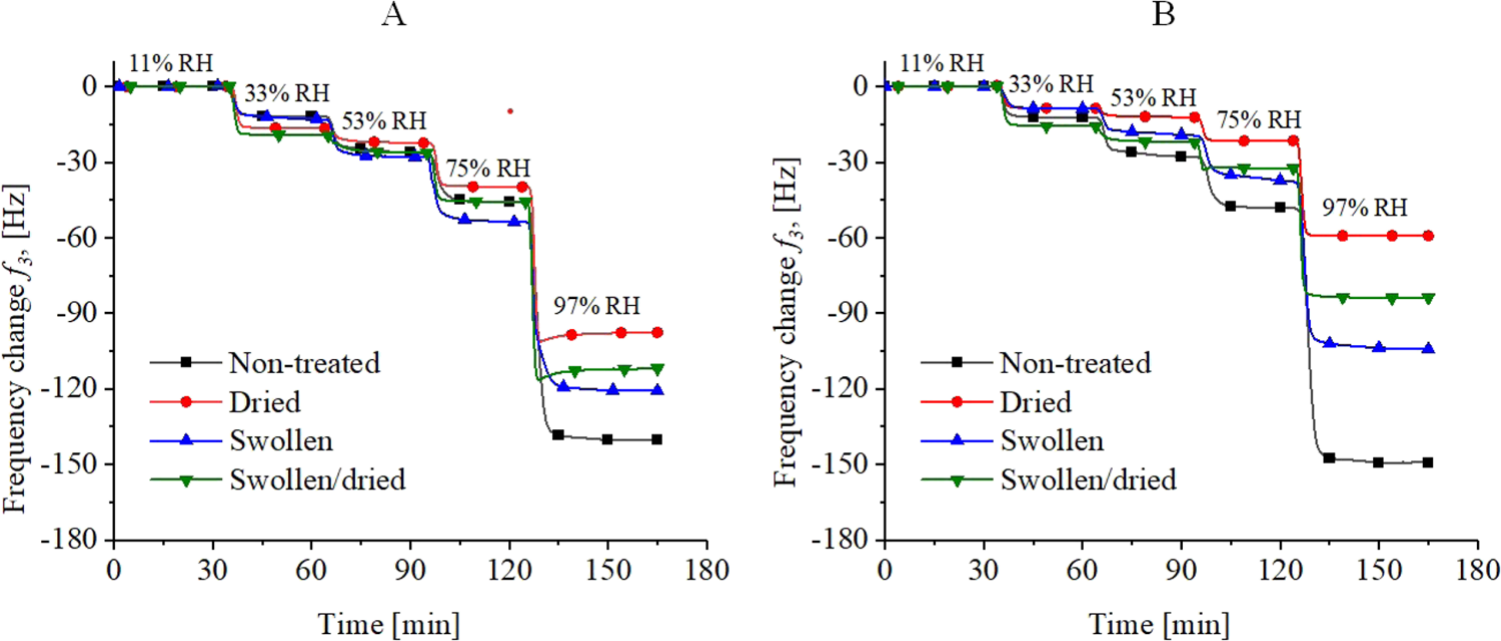
QCM-D data highlighting the change in frequency during water vapor uptake experiments on Cell_A_ (A) and Cell_S_ (B) films at different humidity levels. Changes in the third overtone are shown. Please note that there are hardly any changes in dissipation (Figure S3; Supporting Information) and that the Cell_A_ films feature higher film thickness than Cell_S._ All experiments have been performed on four different films.

In order to validate the results obtained by XRR, a second technique was employed to determine the water vapor uptake capacity of the cellulose films. For this purpose, QCM-D measurements equipped with a humidity module were performed. Since the setup of the QCM-D uses water vapor permeable membranes to adjust RH, higher RH (up to 97%) than in XRR can be obtained. While XRR is a spectroscopic technique, QCM-D exploits gravimetric principles based on the Sauerbrey equation which relates the eigenfrequency of a resonating system to its mass. In more detail, the change in frequency (Δ*f*) of a QCM-D sensor allows for monitoring changes in the film mass thereby providing information on the mass of sorbed water vapor on the surface as well as inside the sample. The films deposited on the QCM sensors had a thickness of 50 ± 6 nm (Cell_A_) and 39 ± 5 nm (Cell_S_), which is in good agreement with XRR given that the substrate is different (oxidized silicon wafer vs gold surface). Exposure of these films to different humidity levels resulted in a negative change in frequency, which correlates with an increase in film mass (Figure 4).

The results of the QCM-D measurements followed the same trends as already shown in the XRR investigations. The dried samples exhibited the lowest water vapor uptake at the different RH for all samples, whereas the differences between the samples were most pronounced at 97%RH. The particular difference between the Cell_A_ and Cell_S_ derived samples was also reflected in the QCM-D measurements. Except for the dried sample, water vapor uptake was nearly the same for all treated and the nontreated films as those determined by the XRR measurements. For both systems, the situation was similar as for the XRR and the swollen/dried films showed a lower water vapor uptake than the swollen films. Even the relative raise in film thickness for the different films was in good agreement with the XRR data. For instance, the nontreated Cell_A_ sample featured an increase of film thickness of 16.4% at 75%RH (compare XRR: 16.1%). However, while trends were represented in a similar manner as in the XRR experiments, for some samples a smaller water vapor uptake is accomplished in the QCM-D studies (Figure S4). After a further increase of the RH up to 97%RH the difference between the nontreated (42.3%) and dried (28.0%) films was even more distinct. The results for the pristine films were comparable to previous findings.^30,32^

**Figure 5:**
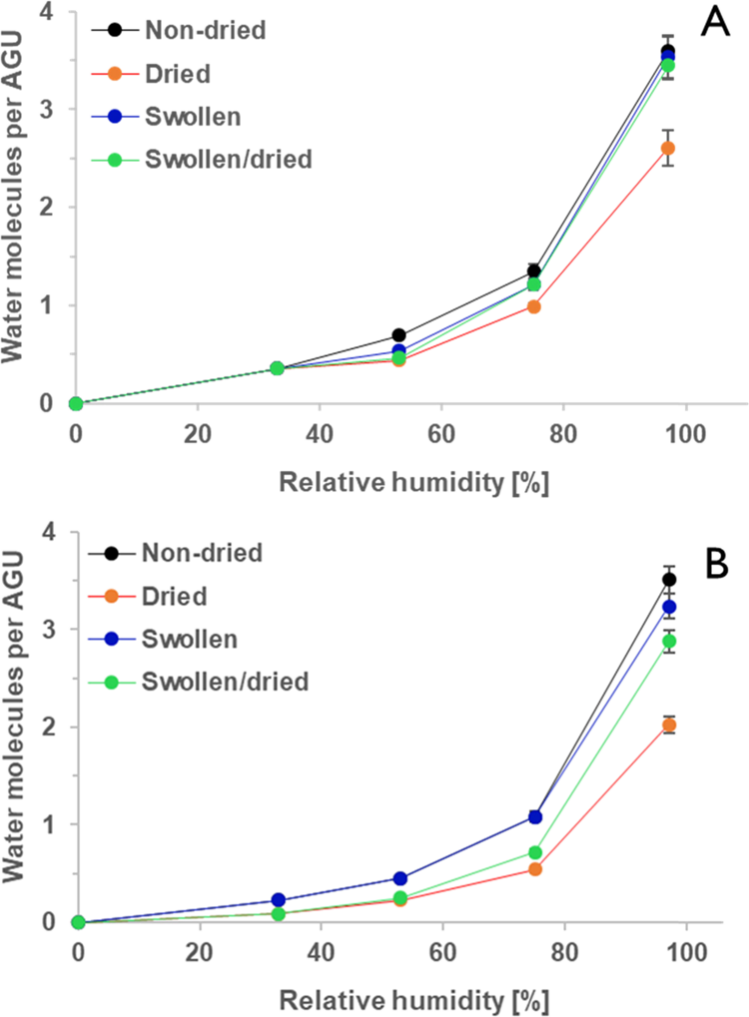
Effect of the RH and applied treatments on water uptake obtained by QCM-D. (A) Cell_A_, (B) Cell_S_. All experiments have been performed on four different films. Note that the standard deviation for the films between 0 and 75% rh is smaller than the dot diameter in the diagram and is therefore not visualized in the figure for readability.

These results can be presented also in terms of the mass of absorbed water per mass of cellulose. This allows for the calculation of the number of water molecules which are embedded per anhydroglucose unit (AGU) by calculating the molar ratio of water and cellulose (Figure 5). The uptake of water vapor into the films at lower humidity levels led to the incorporation of less than one water molecule/AGU. By increasing the humidity to 75%RH, the ratio of water molecules/AGU in nontreated films raised to 1.08 (Cell_S_) and 1.35 (Cell_A_) and at 97% it increased to nearly 3.6 for both nontreated films. This is an interesting finding since earlier reports on liquid water uptake on similar regenerated cellulose thin films concluded that five molecules of liquid water were present at each AGU.^44^ Drying the films reduced the water/AGU ratio, particularly when rather high RH levels were employed (Figure 5).

In addition to information on mass changes, the dissipation module of the QCM allows for interpreting and monitoring changes in the viscoelastic behavior of the films. This is accomplished by determination of dissipated energy at a given overtone, denoted as Δ*D_n_*. One might expect that the incorporation of the water into the film structure will lead to a softening concomitant with increased film viscosity (i.e., reduced elasticity) and a subsequent increase in Δ*D_n_*. Similar to a recent report,^30^ we did not observe any changes in the viscoelastic behavior of the films by QCM-D (Figure S3B, Supporting Information). However, the situation seems to be complex and depends on the order inside the films as shown in another report.^32^ The authors prepared films from TMSC but used two different film preparation methods (spin coating vs Langmuir–Schaefer deposition) that yielded crystalline and amorphous thin films. The crystalline films took up more water vapor than the amorphous ones. On the contrary, the dissipation values for the amorphous films were higher than for the crystalline ones. The authors related that to the incorporation of water into nanopores of the crystalline domains, where the water is strongly bound and cannot act as a plasticizer, thereby restricting viscoelasticity. Therefore, it seems that our films respond more like ordered rigid-like structures that can incorporate water in confined environments. This is supported by our recent findings that the cellulose thin films feature domains with a short-range order. In these domains, the cellulose molecules can be arranged in two different configurations with respect to the surfaces with dimensions of 3 and 6 nm, respectively.^39^ This should be actually an entropically driven process as the water would avoid being destructured (hydrophobic effect) by the ordered cellulose domains as shown recently in seminal works on cellulose nanocrystals.^45^

In addition, the sample preparation in that paper may influence the behavior of the films in terms of viscoelasticity. The authors in that work used a polystyrene (PS)-coated QCM sensor to deposit the TMSC from chloroform solutions. Since chloroform is a very good solvent for PS, this may lead to partial dissolution of the PS accompanied by penetration of TMSC into the PS layer during the spin coating step. Partially phase separated domains may form at the PS/TMSC interface as described in other publications.^46^ Upon regeneration, the interfacial tension may induce the cellulose domain to rearrange resulting in a different viscoelastic behavior than for a film directly deposited on the QCM sensor, while surface properties at the air/cellulose interface should remain unaffected.

In principle, changes in the density of the cellulose films can also be tracked using the QCM-D. Since the mass of water per gram of cellulose has been determined, the changes in density upon water uptake can be easily followed during water vapor uptake. However, the starting density must be set to the one of amorphous cellulose. For all the films, there is a clear trend, namely, that densities significantly decrease with increasing water vapor uptake. The decrease is slightly more pronounced for the Cell_S_ samples. Interestingly, the density of the films is very similar for all the samples at RH levels of 97% (1.30–1.34 g·cm^–3^). A comparison between the densities obtained by XRR and QCM-D is depicted in Table S3, Supporting Information.

The results can be rationalized concerning two major aspects. The first interesting finding was that the very interface between the substrate and the cellulose “bulk” layer is rather different to the bulk layer. Although some functionalized cellulose derivatives have been exploited for monolayer formation,^47−51^ there are only a few studies available which have attempted the generation of neat cellulose monolayers. For these cases, either submonolayers, fractal structures, or open films have been realized.^52,53^ Therefore, the exact nature of such interfaces and their importance for film formation still remains hardly accessible. For all the investigated samples in this paper, the interfacial cellulose layer at the substrate features a thickness of ca. 0.5–0.7 nm. This thickness corresponds to one or two stapled cellulose layers. Probably, the constrained environment (i.e., a smooth, regular, nonswelling, OH rich surface) of the substrate forces the cellulose chains during regeneration into a parallel, flat arrangement with respect to the substrate surface. This interfacial cellulose layer is surprisingly stable and does not vertically extend during exposure to increased humidity levels as shown by XRR. It is mere speculation as to whether either the water molecules are incapable of diffusion to this interfacial layer, or they are incorporated in voids between individual macromolecules within the layer structure. It is evident that this layer must feature a rather good interaction with the hydrophilic silicon oxide substrate via hydrogen bonding which may compete with those of water vapor. We gained weak indications earlier that the biochemistry of this interlayer is different to the “bulk” layer. Some of us noticed that during enzymatic hydrolysis monitored by AFM occasionally an extremely thin layer (<1 nm) of cellulose was left on the silicon substrates. It seemed that the cellulase cocktail was not capable of degrading this part of the cellulose film.^54^ Since in many biological processes interfacial phenomena play a large role, the boundaries to other materials classes are of particular importance for the function of biological systems. In general, the properties of polymer thin films are different in regions near interfaces compared to the bulk. Particularly, the mobility of the polymer chains and the glass transition temperature have been identified as parameters that vary between bulk and interfacial polymer layers. At the substrate interface, polymer chains experience increased *T*_g_ accompanied by reduced chain mobility because of interactions between the surface and the macromolecules.^55−57^

The second aspect concerns the influence of the treatments on the hydration of the cellulose macromolecules. For the nontreated films, 3.6 molecules of water are present per AGU of cellulose for both investigated cellulose films. These values are in excellent agreement with available data on nontreated cellulose thin films which have been prepared the same way as in this study.

In this work, the response of Cell_A_ and Cell_S_ at lower humidity levels and the applied treatments differed to some extent. The results follow a common, rather unexpected trend: the degree of hydration of Cell_A_ is systematically higher for all samples at the same humidity level/treatment than those of Cell_S_. Except for the 97% humidity level, even the nontreated films show differences which are at their most pronounced at 75% RH (1.35 vs 1.08 molecules H_2_O/AGU for Cell_A_ and Cell_S_). This is rather surprising since there is a difference in 20% of water vapor uptake for a material featuring the same chemistry, and similar morphology. Since the molecular weights are very similar, these differences may relate to the preparation procedure (spin coating from THF vs chloroform). The main differences in the preparation are the different vapor pressures of the solvents (190 vs 270 mbar at 20 °C) as well as the concentration of the solution used for spin coating. As shown in an earlier report, the degree of molecular entanglement of TMSC macromolecules is different when different concentrations are used, even when parameters such as viscosity are nearly identical.^58^ While in that study, the degree of entanglement had a large impact on the shape of the obtained materials (semispheres or fibers), here the differences may be related to different orientations of the macromolecules to each other, leading to different types of amorphous films having different arrangements. The existence of short-range orders having domain sizes of ca. 3 to 6 nm definitely influence water uptake into the films.^39^ Furthermore, the different vapor pressures of the employed solvents may contribute to this effect. The necessity to induce a third layer for the description of the Cell_A_ films, also point the distinction of the film structures between Cell_A_ and Cell_S_. Nevertheless, it is intriguing that these, on a first glance, subtle differences lead to such distinct water vapor uptake phenomena. Besides the different behavior of the films in terms of preparation conditions, the impact of the treatments on the amount of water molecules/AGU is instructive. Nontreated and swollen films show a similar behavior at lower humidity levels, whereas an additional drying step after the swelling reduces the water incorporation into the films. Drying directly after preparation of the films largely reduces the amount of water molecules/AGU at high and medium humidity levels.

In order to track changes on the pore level and the uptake of water vapor into the cellulose network in the nanometer scale, we performed GI-SAXS for the Cell_A_ series at controlled humidity. The vertical cuts revealed a shift of the fringes to lower *q* values and approved the swelling of the samples (Figure S6, Supporting Information). Figure S7 (Supporting Information) depicts the results of the integration along the horizontal cuts obtained for the samples at different humidity levels (typical horizontal cut is shown in Figure S8, Supporting Information). Even on a qualitative basis, it can be already seen that the trends observed by the other methods are reflected in parts in the GI-SAXS measurements as well. The nontreated sample shows the strongest change in the nanoscale supramolecular structure upon increasing humidity levels, while the dried samples had a lower response to humidity. These rather qualitative statements can be transformed into more quantitative assessments by analyzing the data according to the models theoretically described in the Materials and Methods section. These models use as basis a single macromolecule approximated by a simple Hamouda approach^38^ for an infinitely long cylinder (*R*_g_ fixed at 1 nm for the cross section, *s* = 1 and *q* = 4). For the description of the assembly of the cellulose macromolecules, the macromolecule–macromolecule correlations have been derived from a simplified interaction term consisting of the Lorentz peak at the mean distance of the molecules. The peak intensity and peak width therefore correspond to the degree of order in the supramolecular structure while the position of the peak determines the mean distance between the macromolecules.

The data show that for all except the dried samples the incorporation of water into the pores of the films leads to decreased relative peak intensities (Table S5; in a.u). This means that air in nanosized pores is replaced by water at increasing humidity levels, thereby reducing the X-ray contrast in the films. It should be noted that the absolute values of the peak intensities should not be directly compared but only the relative changes. According to the GI-SAXS data for the dried films, the vapor does not replace air at elevated humidity levels. A potential explanation could be the intercalation of water directly into the supramolecular structure. This may originate by rearrangements in the film structure, as shown recently.^41^

## Conclusions

Despite many attempts in the past to unravel interactions of water and cellulose, the rather high complexity makes it rather arduous to assess the basic underlying mechanisms. This is particularly relevant for “real” cellulosic samples where complex pre- and post-treatments are regularly applied to realize certain material characteristics. The multiscale hierarchical structure of natural fibers adds to the complexity as well. However, even for rather simple model film approaches such as mixed crystalline and amorphous cellulose films, unexpected behavior of the cellulose materials is observed. Even if the complexity is further reduced by investigating mostly amorphous cellulose films like in the present study, the preparation conditions largely affect the interaction of the films with water vapor at different humidity levels. As determined by nanoindentation experiments carried out using AFM instrumentation, the stiffness of the two respective films is different which may contribute to the different behavior of the two films. However, there are also distinct differences in the film structures. While for both films an interfacial layer to the substrate was introduced for the evaluation of the XRR data, for the Cell_S_ sample a third layer at the cellulose/air interface was required to achieve a satisfactory fit. Such differences certainly stem from the different preparation procedures since the used solvents exhibit rather different vapor pressures. The employed physical treatments for the thin films give rise to similar processes that occur in macroscopic samples. Similar to liquid water, water vapor incorporation decreases when the samples have been dried at elevated temperatures. This behavior becomes particularly pronounced at elevated humidity levels, where the amount of water molecules/AGU can be significantly reduced from 3.6 to 2.6 (Cell_A_)/2.0 (Cell_S_) for the differently prepared samples at 97% RH. It seems that water vapor is involved in its incorporation into the supramolecular structure as shown by GI-SAXS and XRR.

## Supporting Information Available

Supplementary data on roughness, XRR, QCM-D, and GI-SAXS and detailed information derived from XRR, QCM-D and GI-SAXS (PDF)
