# Supplementary material for: Humidity Response of Cellulose Thin Films
Source: Biomacromolecules. 2022 Feb 28;23(3):1148–57. doi: 10.1021/acs.biomac.1c01446 (PMC8924868; doi:10.1021/acs.biomac.1c01446)
Supplement: Supplementary file 1 — bm1c01446_si_001.pdf [file bm1c01446_si_001.pdf]

Humidity Response of Cellulose Thin Films

*David Reishofer[‡], Roland Resel , Jürgen Sattelkow[§], Wolfgang J. Fischer[‡], Katrin Niegelhell[‡], Tamilselvan Mohan[$], Karin Stana Kleinschek[$], Heinz Amenitsch[◊], Harald Plank[§], Tekla Tammelin[¥], Eero Kontturi[‖ *] and Stefan Spirk[‡*]*

[‡]Institute of Bioproducts and Paper Technology, Graz University of Technology, Inffeldgasse 23, 8010 Graz, Austria

Institute for Solid State Physics, Graz University of Technology, Petersgasse 16, 8010 Graz, Austria.

[§]Graz University of Technology, Institute for Electron Microscopy and Nanoanalysis, Steyrergasse 17, 8010 Graz, Austria.

[$]Institute of Chemistry and Technology of Biobased Systems, Graz University of Technology, Stremayrgasse 9, 8010 Graz, Austria.

[◊]Institute for Inorganic Chemistry, Graz University of Technology, Stremayrgasse 9, 8010 Graz, Austria.

Electronic Supporting Information

[¥]High Performance Fibre Products, VTT Technical Research Center of Finland Ltd, FI-02044 VTT, Espoo, Finland.

[‖]Department of Department of bioproducts and Biosystems,, School of Chemical Technology, Aalto University, 02150 Espoo, Finland.

Corresponding Author

* E-mail: eero.kontturi@aalto.fi, Phone:

*E-mail: stefan.spirk@tugraz.at, Phone: +43-316-873-32284

Members of the European Polysaccharide Network of Excellence (EPNOE).

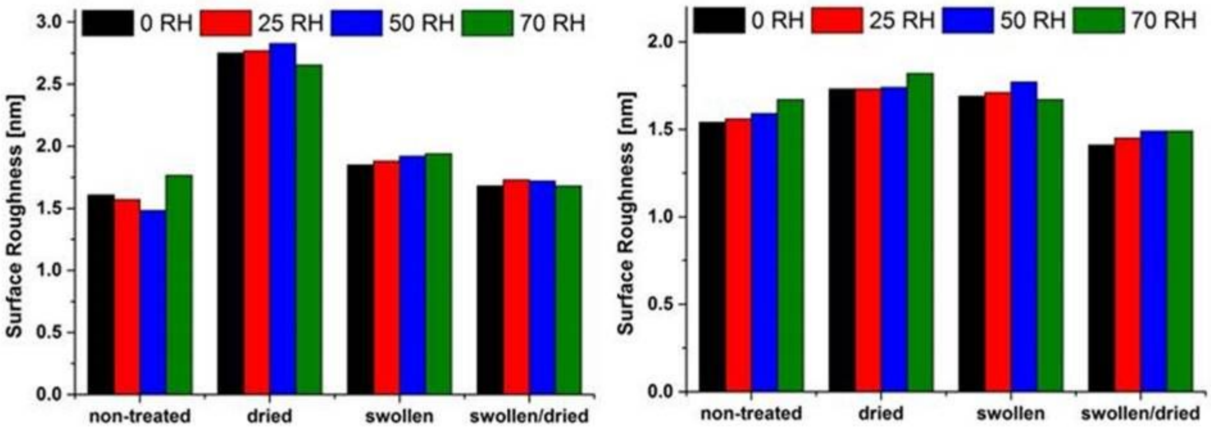

Figure S1. Surface roughness of $Cell_A$ (left) and $Cell_S$ (right) samples determined by XRR during water vapor uptake experiment. Note the different scale of the two graphs.

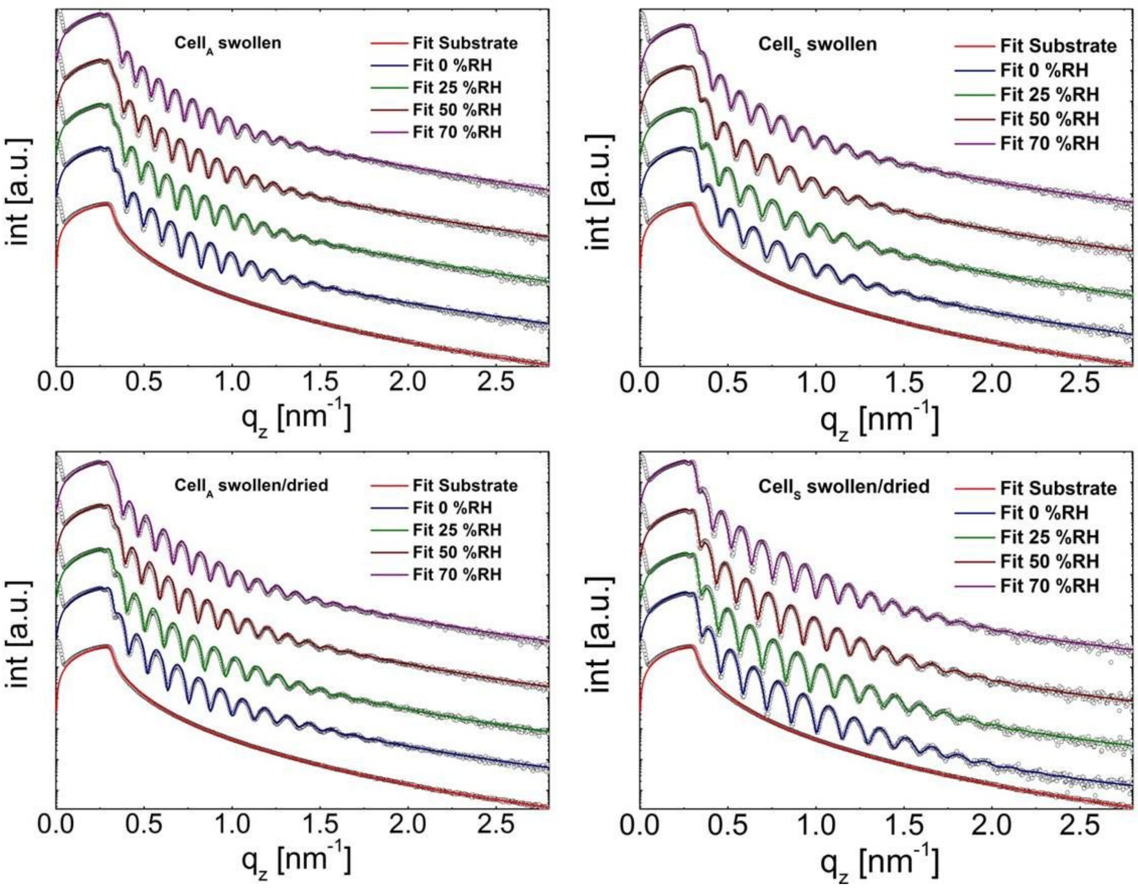

Figure S2. XRR curves and corresponding layer fit of the two different cellulose films (Cell$_A$, left column; Cell$_S$, right column) samples after different treatments.

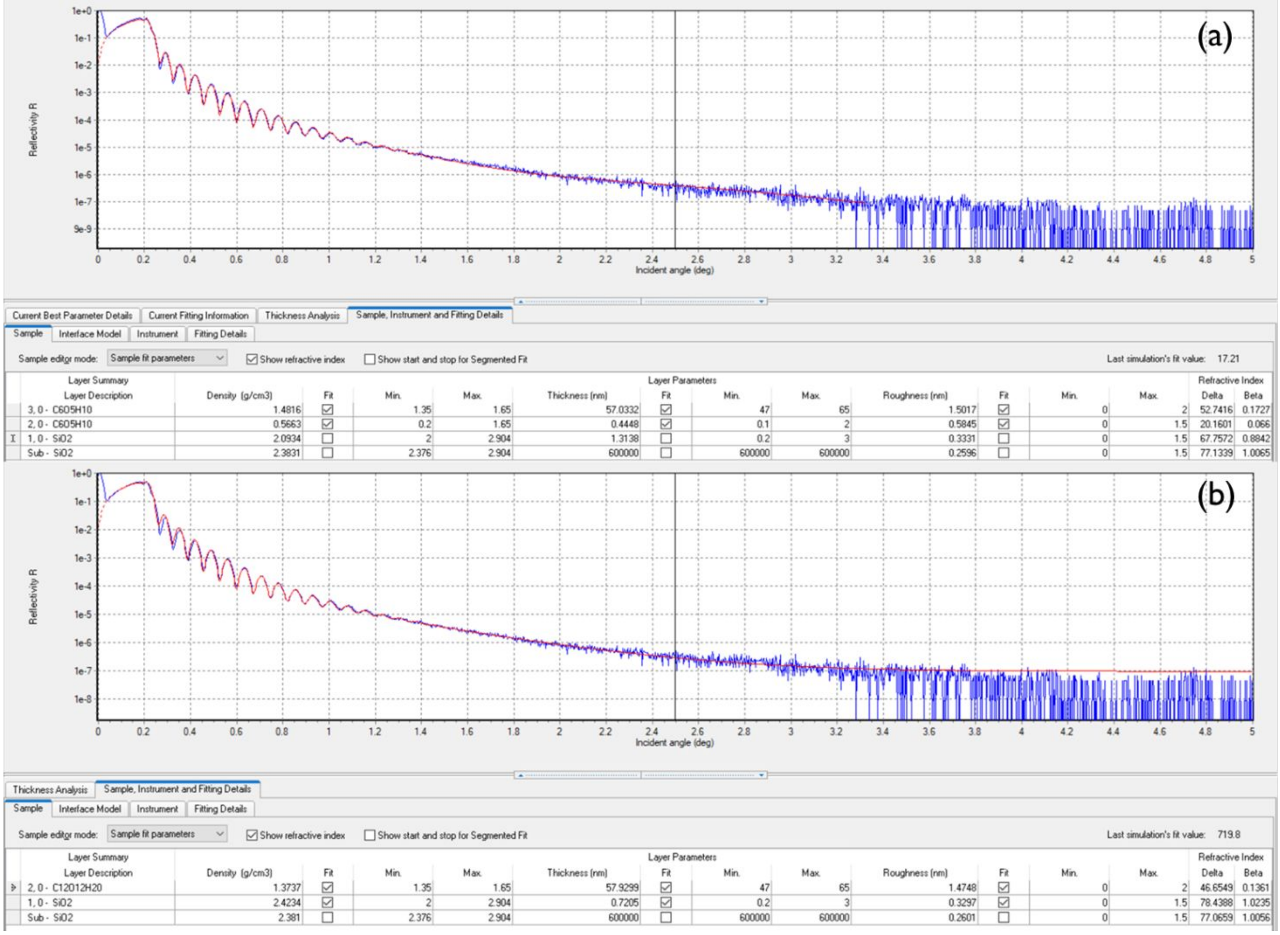

Figure S3. XRR measured data and fits of CellA (non-treated) at 50% relative humidity demonstrating the influence of the chosen model approach using a) a two layer fit for the cellulose film, b) using a single layer fit for the cellulose film. For b), the values do not make physically sense and have a poorer fitting quality. Attempts to get better fits for a single cellulose layer fit were unsuccessful.

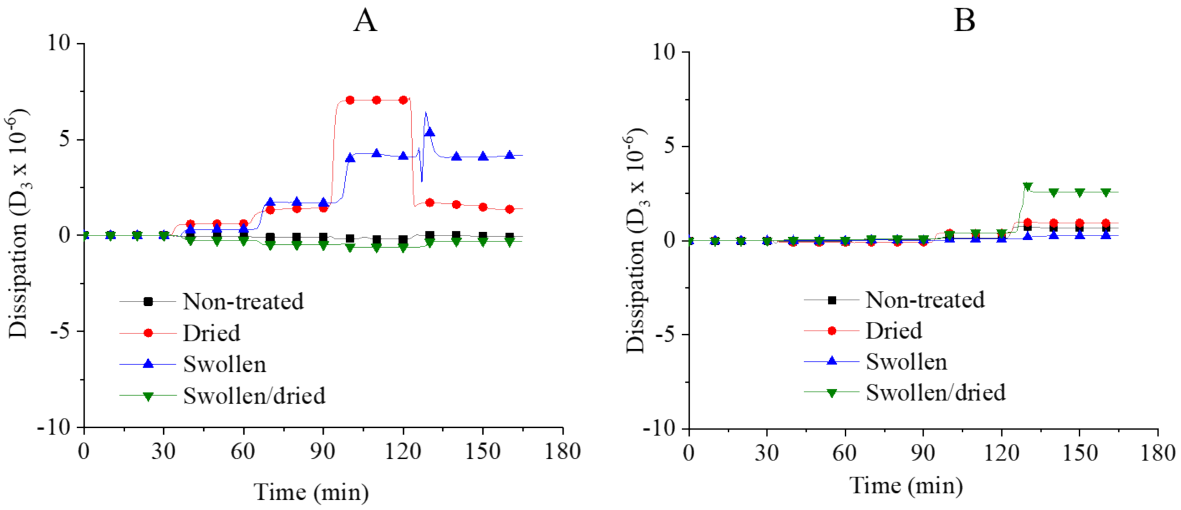

Figure S4. QCM-D data highlighting the change in dissipation during water vapor uptake experiments on $Cell_A$ (A) and $Cell_S$ (B) films at different humidity levels and treatments. Changes in the ΔD_3 are shown.

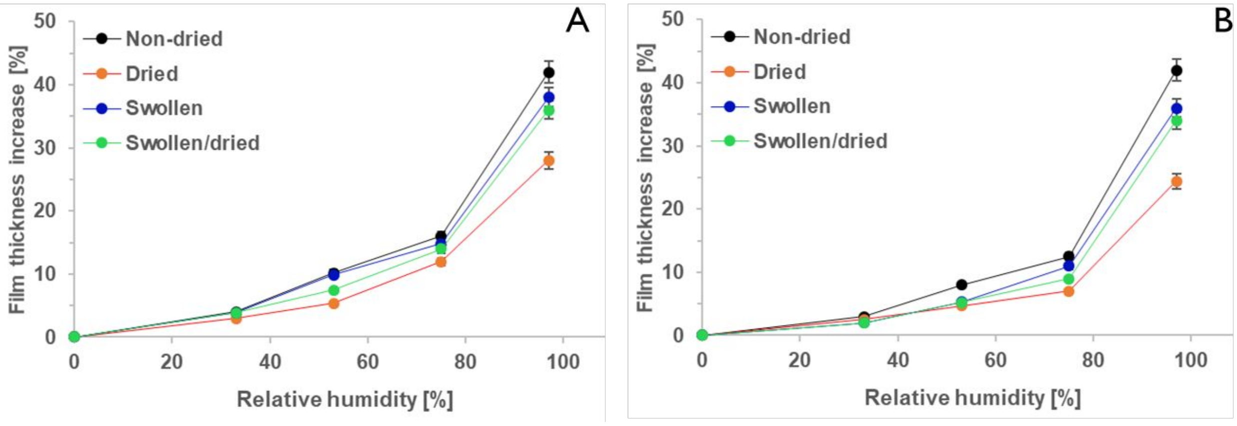

Figure S5. Film thickness increase at different humidity levels determined by QCM-D A) $Cell_A$ samples B) $Cell_S$ samples. Note that the standard deviation for the films between 0 and 75% rh is smaller than the dot diameter in the diagram and is therefore not visualized in the figure for readibility.

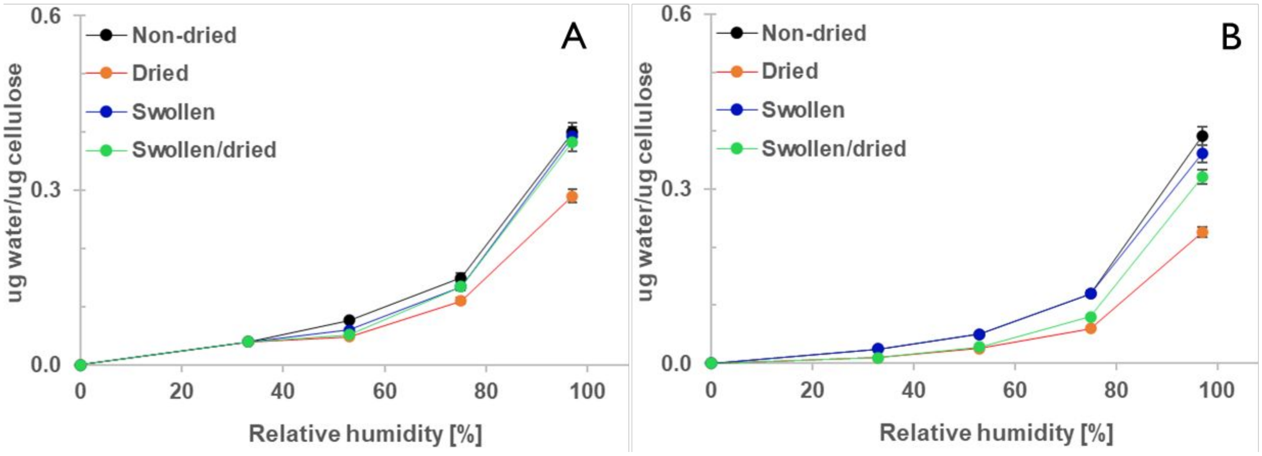

Figure S6. Comparison of the hydration of cellulose in dependence of the relative humidity and applied treatments obtained by QCM-D. A: CellA, B: CellS. All experiments have been performed on four different films. Note that the standard deviation for the films between 0 and 75% rh is smaller than the dot diameter in the diagram and is therefore not visualized in the figure for readibility.

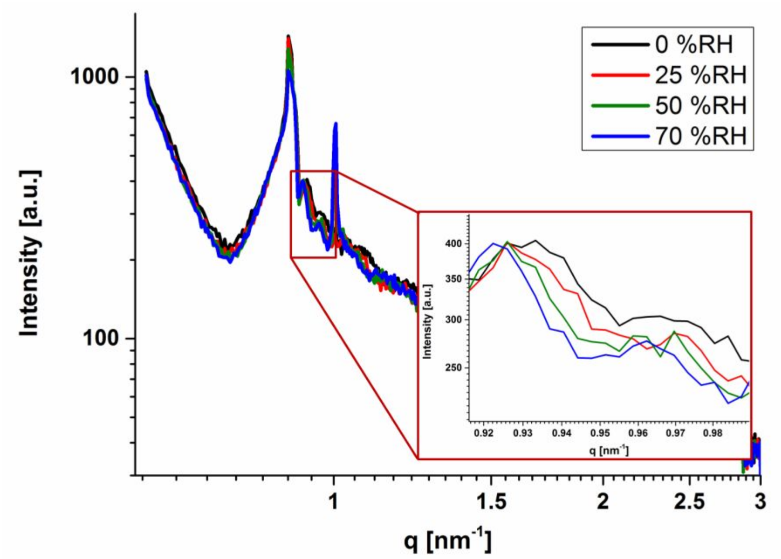

Figure S7. Results of GI-SAXS (vertical cut) for $Cell_S$ non-treated at different %RH values.

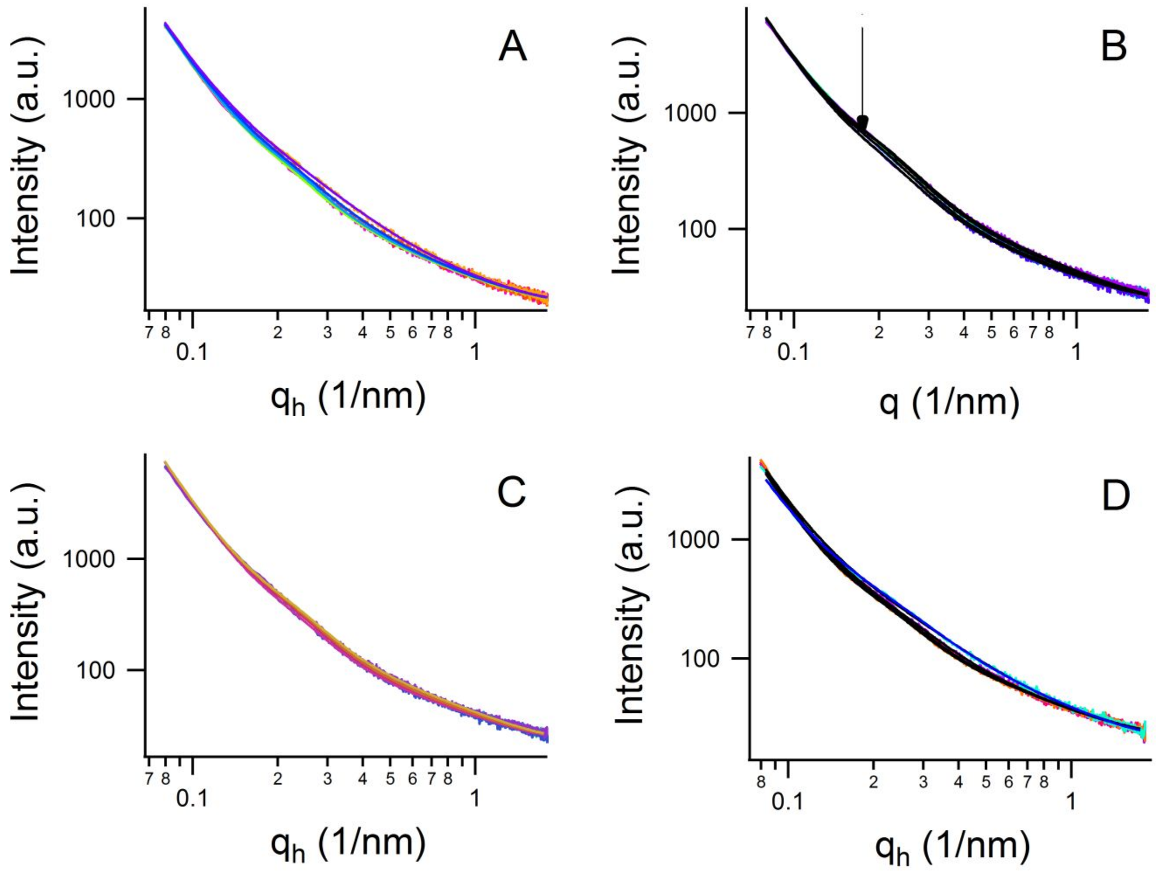

Figure S8. Horizontal cuts of GI-SAXS pattern for $Cell_S$ before and after the various treatments at humidity levels 0, 25, 50, 70%RH (colors in bracket). A: non-treated, B: swollen, C: swollen followed by drying at 105 ˚C for 3 hours, D: drying at 105 ˚C for 3 hours. The correlation peak of the cellulose macromolecules/porous network is indicated with an arrow (B).

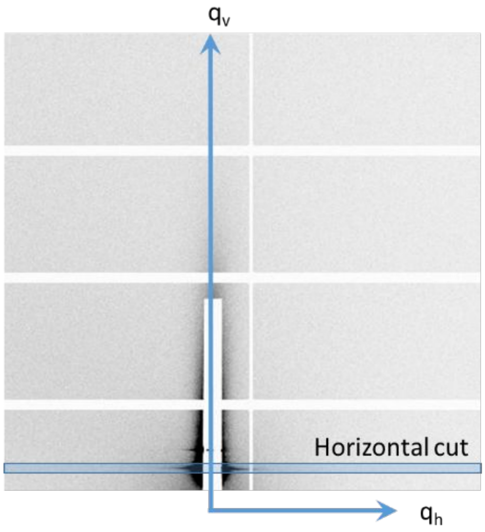

Figure S9. Typical example of an obtained GISAXS pattern with the indication of the horizontal cut used for the further data analysis.

Table S1. XRR results of $Cell_A$ samples (two-layer fit; three layer fit for swollen sample): Fitting error < 0.3% for all samples.

$Cell_A$	Layer 1			Layer 2			Layer 3			Total Thickness	
% RH	Nm		g/cm³	nm		g/cm³	Nm		g/cm³	nm	%
non-treated	Thickness	Roughness	Density	Thickness	Roughness	Density				Thickness	Thickness
0	0.6	0.2	0.92	51.9	1.6	1.45				52.5	0.0
25	0.6	0.2	0.90	54.1	1.6	1.43				54.7	4.3
50	0.5	0.3	0.67	56.7	1.5	1.48				57.2	9.0
70	0.5	0.3	0.66	60.8	1.8	1.41				61.3	16.1
dried	Thickness	Roughness	Density	Thickness	Roughness	Density				Thickness	Thickness
0	0.6	0.3	1.10	53.8	2.8	1.49				54.4	0.0
25	0.5	0.2	1.04	56.0	2.8	1.48				56.5	3.8
50	1.0	0.5	1.06	57.4	2.8	1.44				58.4	7.2
70	1.3	0.5	1.04	59.8	2.7	1.40				61.1	12.2
swollen	Thickness	Roughness	Density	Thickness	Roughness	Density	Thickness	Roughness	Density	Thickness	Thickness
0	0.4	0.3	0.99	47.0	3.1	1.45	4.6	1.6	1.17	52.0	0.0
25	0.4	0.3	0.96	48.9	3.1	1.43	4.9	1.6	1.15	54.2	4.4
50	0.4	0.3	0.95	51.0	3.2	1.42	5.4	1.7	1.11	56.8	9.2
70	0.4	0.3	0.94	52.9	3.6	1.39	6.2	1.7	1.10	59.5	14.6
swollen dried	Thickness	Roughness	Density	Thickness	Roughness	Density				Thickness	Thickness
0	0.6	0.2	0.92	47.8	1.7	1.44				48.4	0.0
25	0.6	0.3	0.91	50.0	1.7	1.47				50.6	4.5
50	0.6	0.2	0.89	52.3	1.7	1.45				52.9	9.4
70	0.6	0.2	0.86	55.1	1.7	1.42				55.7	15.2

Table S2. XRR results of $Cell_S$ samples (three-layer fit). Fitting error < 0.3% for all samples.

$Cell_S$	Layer 1			Layer 2			Layer 3			Total Thickness	
**% RH**	**Nm**		**g/cm³**	**nm**		**g/cm³**	**nm**		**g/cm³**	**nm**	**%**
**non-treated**	**Thickness**	**Roughness**	**Density**	**Thickness**	**Roughness**	**Density**	**Thickness**	**Roughness**	**Density**	**Thickness**	**Thickness**
**0**	0.6	0.3	0.81	40.4	3.0	1.50	4.2	1.5	1.15	45.2	0.0
**25**	0.6	0.3	0.79	42.2	3.1	1.50	4.3	1.6	1.14	47.1	4.3
**50**	0.6	0.3	0.75	44.5	3.1	1.48	4.3	1.6	1.12	49.4	9.4
**70**	0.6	0.2	0.72	46.7	3.3	1.48	5.0	1.7	1.14	52.3	16.0
**dried**	**Thickness**	**Roughness**	**Density**	**Thickness**	**Roughness**	**Density**	**Thickness**	**Roughness**	**Density**	**Thickness**	**Thickness**
**0**	0.6	0.2	0.80	40.4	2.7	1.47	4.2	1.7	1.10	45.2	0.0
**25**	0.6	0.2	0.76	41.9	2.3	1.45	4.3	1.7	1.08	46.8	3.7
**50**	0.7	0.2	0.83	43.9	1.8	1.39	4.2	1.7	1.05	48.7	7.9
**70**	0.6	0.2	0.86	46.4	2.2	1.40	4.7	1.8	1.01	51.7	14.4
**swollen**	**Thickness**	**Roughness**	**Density**	**Thickness**	**Roughness**	**Density**	**Thickness**	**Roughness**	**Density**	**Thickness**	**Thickness**
**0**	0.5	0.3	0.84	36.5	2.9	1.45	4.6	1.7	0.97	41.6	0.0
**25**	0.5	0.2	0.87	38.3	2.8	1.45	4.6	1.7	0.99	43.3	4.2
**50**	0.5	0.3	0.84	40.5	3.0	1.46	4.6	1.8	1.00	45.7	9.7
**70**	0.7	0.2	0.75	43.1	2.3	1.40	4.7	1.7	1.08	48.5	16.7
**swollen dried**	**Thickness**	**Roughness**	**Density**	**Thickness**	**Roughness**	**Density**	**Thickness**	**Roughness**	**Density**	**Thickness**	**Thickness**
**0**	0.5	0.2	0.93	36.4	2.6	1.47	4.7	1.4	1.19	41.6	0.0
**25**	0.5	0.3	0.93	37.5	3.3	1.50	5.3	1.5	1.23	43.4	4.3
**50**	0.5	0.2	0.92	39.0	3.7	1.53	5.9	1.5	1.27	45.4	9.2
**70**	0.5	0.2	0.92	41.5	3.9	1.54	6.2	1.5	1.28	48.2	16.0

Table S3. Comparison of mass densities obtained by XRR and QCM-D. Please note the slight differences in humidity levels for both methods. The density of the QCM-D films was set to the value of amorphous cellulose (1.48 g·cm^{-3}). Changes induced by increasing humidity levels are related to this starting density.

Relative Humidity [%RH]		$Cell_A$		$Cell_S$	
XRR	QCM-D	XRR	QCM-D	XRR	QCM-D
non-treated		Density [g*cm³]			
0	11	1.45	1.48[a]	1.45	1.48
25	33	1.45	1.47	1.43	1.47
50	53	1.43	1.44	1.43	1.44
70	75	1.43	1.40	1.41	1.39
	97		1.30		1.31
dried					
0	11	1.42	1.48[a]	1.49	1.48
25	33	1.40	1.47	1.48	1.47
50	53	1.34	1.44	1.44	1.45
70	75	1.35	1.40	1.40	1.41
	97		1.32		1.34
swollen					
0	11	1.41	1.48[a]	1.41	1.48
25	33	1.38	1.47	1.39	1.47
50	53	1.37	1.41	1.38	1.44
70	75	1.34	1.40	1.35	1.43
	97		1.31		1.33
swollen/dried					
0	11	1.44	1.48[a]	1.44	1.48
25	33	1.42	1.47	1.47	1.47
50	53	1.39	1.44	1.45	1.45
70	75	1.38	1.41	1.42	1.42
	97		1.33		1.34

Table S4. Relative humidities of each saturated salt solution and water used in the QCM-D relative humidity cycles.[1]

Salt	Relative humidity (RH)
$LiCl_{(aq)}$	11
$MgCl_{2(aq)}$	33
$Mg(NO_3)_{2(aq)}$	53
$NaCl_{(aq)}$	75
$K_2SO_{4(aq)}$	97
$MilliQ_{(l)}$	100

Table S5. Overview of the main fit results of the horizontal cuts of the GISAXS experiments. The quantities have been defined in Materials and Methods.

$Cell_S$	0 %RH (dry N_2)		25 %RH		50 %RH		70 %RH	
non-treated	Value	STDV	Value	STDV	Value	STDV	Value	STDV
i0 (a.u.)	14.73	0.16	14.44	0.09	14.47	0.01	13.60	0.89
x_peak (1/nm)	0.13	0.02	0.18	0.01	0.21	0.01	0.21	0.02
	0 %RH (dry N_2)		25 %RH		50 %RH		70 %RH	
dried	Value	STDV	Value	STDV	Value	STDV	Value	STDV
i0 (a.u.)	23.50	0.25	22.60	0.12	22.63	0.11	23.81	1.43
x_peak (1/nm)	0.16	0.05	0.17	0.01	0.18	0.01	0.20	0.02
	0 %RH (dry N_2)		25 %RH		50 %RH		70 %RH	
swollen	Value	STDV	Value	STDV	Value	STDV	Value	STDV
i0 (a.u.)	27.83	0.10	25.16	0.01	22.54	0.10	20.97	1.72
x_peak (1/nm)	0.19	0.01	0.18	0.01	0.19	0.01	0.19	0.02
	0 %RH (dry N_2)		25 %RH		50 %RH		70 %RH	
swollen+dried	Value	STDV	Value	STDV	Value	STDV	Value	STDV
i0 (a.u.)	29.48	0.10	26.57	0.10	25.67	0.09	24.12	1.81
x_peak	0.20	0.01	0.20	0.01	0.20	0.01	0.20	0.02

[1] L. Greenspan, *J. Res. Natl. Bureau Stand.* **1977**, *81*, 89.
